# A case report: Cutaneous leishmaniasis mimicking leprosy in an immunocompetent young adult: A diagnostic and therapeutic challenge in the Old World

**DOI:** 10.1016/j.jdcr.2025.10.077

**Published:** 2025-12-06

**Authors:** Rahaf Albajori, Hala AlHader, Kinda ALshawa

**Affiliations:** Department of Dermatology, Damascus University Dermatology Hospital, Damascus, Syria

**Keywords:** atypical presentation, diagnostic challenge, diffuse cutaneous leishmaniasis, immunocompetent host, leonine facies, lepromatous leprosy, young adult

## Introduction

Leishmaniasis encompasses a spectrum of chronic infections in humans and several animal species. It is caused by over 20 species of Leishmania, flagellated protozoans belonging to the order Kinetoplastidae. Transmission is via the bite of infected female sandflies from the genera Phlebotomus and Lutzomyia. The disease has a worldwide distribution, affecting millions of people in South America, the Mediterranean basin, and parts of Asia and Africa. There are 4 major clinical patterns: (1) cutaneous, which is restricted to the skin and is seen more often in the Old World; (2) mucocutaneous, which affects both the skin and mucosal surfaces and occurs almost exclusively in the New World; (3) diffuse cutaneous, which occurs mainly in the New World; and (4) visceral, which affects the organs of the mononuclear phagocyte system, for example, liver and spleen.^1^

According to the World Health Organization, over 90% of cutaneous infections with Leishmania occur in the Middle East (Afghanistan, Algeria, Iran, Iraq, Saudi Arabia, and Syria) and South America (Brazil, Peru, and Colombia).^2^

Diffuse cutaneous leishmaniasis is most closely associated with L. amazonensis, which is considered a New World species.^3^

Given the epidemiological overlap between leishmaniasis and other dermatological conditions, including leprosy, differential diagnosis remains a significant challenge. While Syria is not classified as an endemic area for leprosy according to World Health Organization reports,^4^ several suspected cases have been mentioned without formal documentation, emphasizing the need for accurate diagnostic approaches in nonendemic settings.

## Case presentation

A 19-year-old immunocompetent male from the countryside of Aleppo, Syria, presented with generalized, well-defined infiltrating papules, plaques, and nodules, with leonine facial appearance with eyebrow alopecia, bilateral auricular edema, and infiltration since 2022.

**Fig 1:**
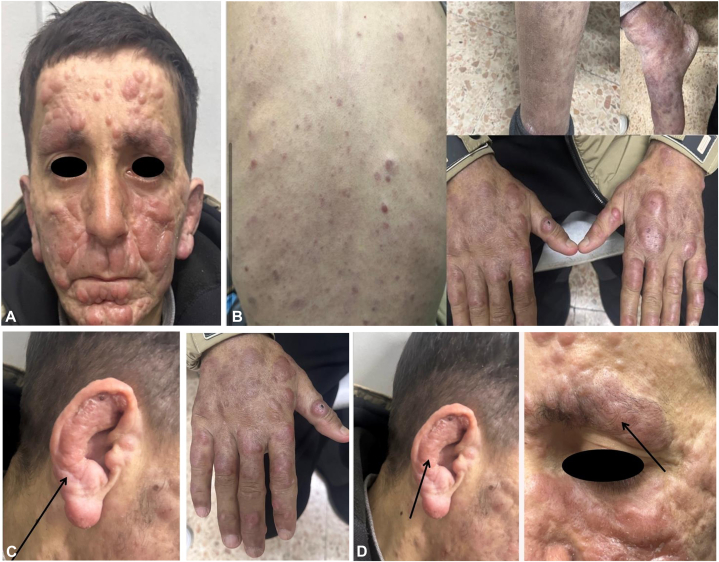
Clinical presentation showing leonine facies **(A)** and widespread infiltrated lesions **(B-D)**. The *black arrows* highlight selected infiltrated lesions in certain affected areas. They are used for visual emphasis only and do not indicate any diagnostic or clinical significance beyond the other lesions.

The condition initially manifested as a single nodule on the face, which gradually expanded Fig 1. Given the endemic nature of cutaneous leishmaniasis, the patient was initially treated with cryotherapy using liquid nitrogen, but the lesions continued to spread. Subsequently, intralesional antimonial injections were administered, followed by systemic antimonial therapy, both without clinical response. This unresponsiveness, along with the leonine facial appearance, initially raised suspicion of lepromatous leprosy, prompting further investigations.

Due to the persistent progression of the lesions, the patient sought medical evaluation at our clinic. The presentation raised suspicion of leprosy, prompting further diagnostic investigations.

Laboratory tests revealed:

Mild anemia (Hb: 9.7 g/dL, Hct: 32%) and elevated C-reactive protein (23 mg/L), suggesting an underlying inflammatory process. Immunoglobulin profiling showed increased IgG and IgM levels (4200 mg/dl and 2200 mg/dl, respectively), while HIV and tuberculin tests were negative.

Normal findings in ultrasound imaging of the liver, gallbladder, spleen, kidneys, and peritoneum.

Negative tuberculin skin test and HIV screening Table I.

**Table I:** Laboratory findings and systemic investigations summarizing organ function and excluding differential diagnoses

Parameter	Value	Unit
HG	9.7	g/dl
HCT	32%	
CRP	23	mg/dl
RBC	3.7	× 10^6^/μL
WBC	7.2	× 10^3^/μL
NEUT	61%	
LYM	21%	
MONO	16%	
PLT	220	× 10^3^/μL
Na	135	mmol/L
K	4.3	mmol/L
Mg	1,9	mg/dl
Glu (FAST)	70	mg/dl
Cr	1	mg/dl
Ur	24	mg/dl
ALT	20	U/L
AST	27	U/L
TG	32	mg/dl
CHOL	53	mg/dl
IgG	4200	mg/dl
IgM	2200	mg/dl
IgA	580	mg/dl
IgE	46	IU/ml
Tuberculin skin test	Negative	___
Toxoplasmosis Ab. IgG	0,5	___
Toxoplasmosis Ab. IgM	0,2	___
HIV test	Negative	___

*ALT*, Alanine aminotransferase; *AST*, aspartate aminotransferase; *CHOL*, cholesterol; *Cr*, creatinine; *CRP*, C-reactive protein; *Glu Fast*, fasting glucose; *HCT*, hematocrit; *HG*, hemoglobin; *IgA*, immunoglobulin A; *IgE*, immunoglobulin E; *IgG*, immunoglobulin G; *IgM*, immunoglobulin M; *K*, potassium; *Lym*, lymphocytes; *Mg*, magnesium; *MONO*, monocytes; *Na*, sodium; *Neut*, neutrophils; *PLT*, platelets; *RBC*, red blood cells; *TG*, triglyceride; *Ur*, urea; *WBC*, white blood cells.

**Fig 2:**
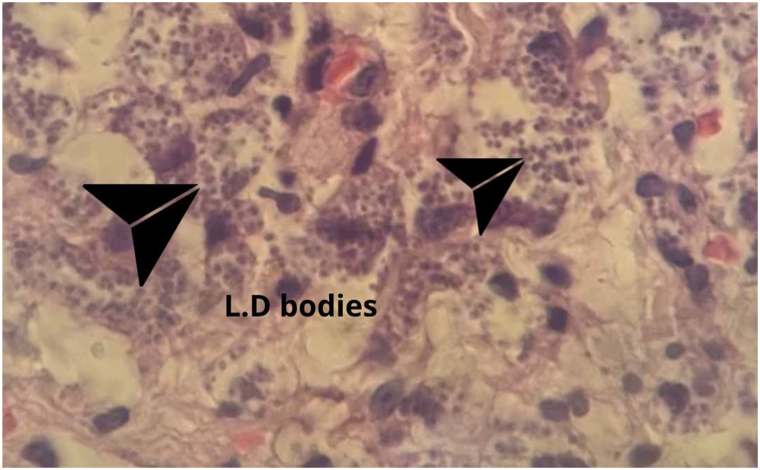
Histopathologic image of a skin biopsy from the right forearm (H&E stain), showing foam cells filled with Leishman-Donovan bodies and granulomatous formations. Extracellular organisms are also visible.

**Fig 3:**
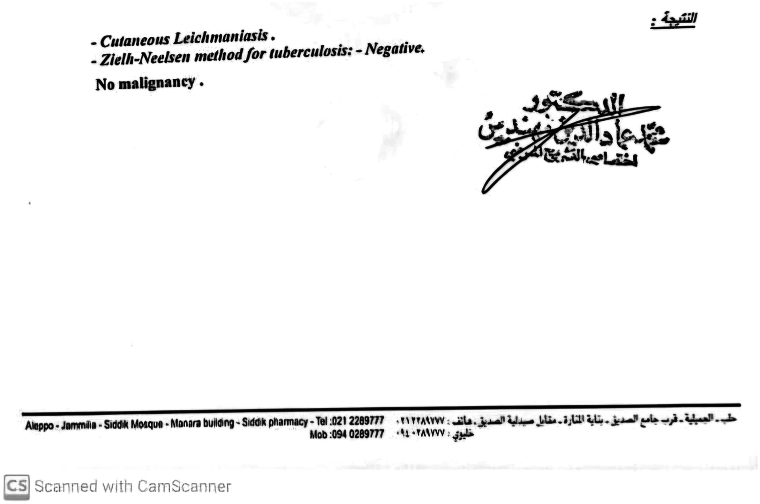
Laboratory report confirming a negative Ziehl-Neelsen stain, ruling out acid-fast bacilli infections.

Upon histopathological examination, biopsy analysis confirmed the presence of Donovan bodies (Fig 2), with negative Ziehl-Neelsen staining (Fig 3), establishing the final diagnosis of diffuse cutaneous leishmaniasis.

## Discussion

Aleppo is endemic for cutaneous leishmaniasis, typically presenting as papules in exposed areas. Diagnosis usually relies on Giemsa-stained direct smear, eliminating the need for polymerase chain reaction (PCR) of cutaneous lesion samples to detect *Leishmania* DNA, or parasite typing.

Leonine facies, eyebrow alopecia, and lesion progression despite standard therapy raised suspicion of lepromatous leprosy. The possibility of dual infection was considered, prompting further investigation.

To investigate the possibility of co-infection, traditional staining techniques were employed. Although Wade staining was initially requested, it was unavailable, necessitating reliance on Ziehl-Neelsen staining, which has lower sensitivity for Mycobacterium leprae. Advanced molecular assays such as PCR targeting M. leprae and Leishmania species could not be performed due to technical limitations, further highlighting the diagnostic challenges in resource-limited settings.

PCR-based detection of M. leprae has demonstrated high sensitivity and specificity, particularly in paucibacillary cases and in regions classified as nonendemic, such as Syria. Its ability to detect minimal bacillary DNA makes it a valuable tool for ruling out co-infection and confirming atypical presentations. In contrast, PCR for Leishmania may exhibit lower specificity in Old World settings due to the widespread prevalence of cutaneous leishmaniasis. However, its utility lies in species-level typing, which is crucial for excluding the possibility of New World variants presenting in Old World regions, especially in cases with atypical clinical or histopathological features.

Histopathological analysis was pivotal in confirming diffuse cutaneous leishmaniasis and excluding leprosy co-infection, based on the absence of characteristic features (Fig 2).

**Fig 4:**
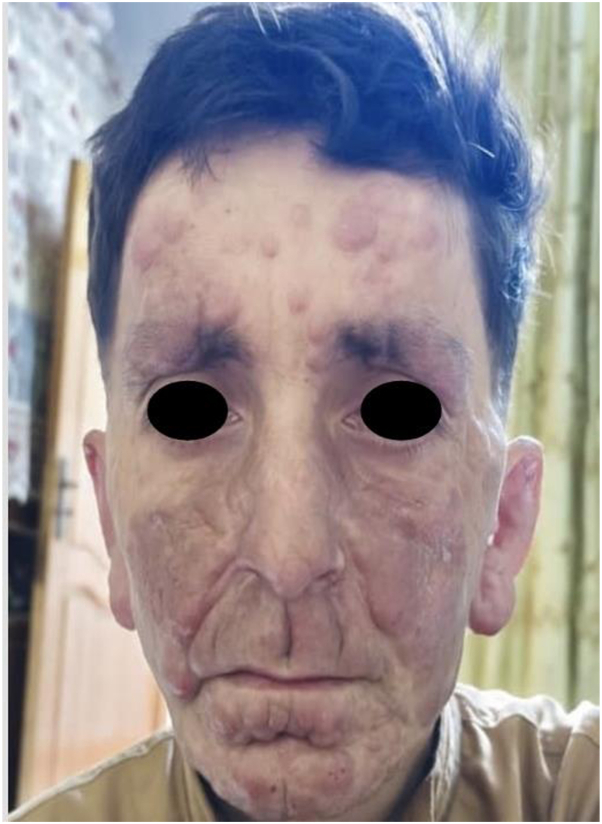
Clinical improvement observed 1 week after Amphotericin B treatment, compared to the initial presentation (Fig 1).

Following exclusion of leprosy, systemic amphotericin B was initiated as a second-line treatment, given the poor response to conventional antimonial therapy. Initial clinical improvement was observed and is illustrated in Fig 4; however, the response plateaued in subsequent sessions, with no further progress. This limited and nonsustained improvement aligns with previous reports describing poor therapeutic outcomes in New World diffuse cutaneous leishmaniasis, particularly with amphotericin B. Although initial responses have been documented, recurrence within months is common, and no data exist regarding the efficacy of lipid formulations in such cases. These findings underscore the importance of species-level identification, as treatment response varies significantly across Leishmania species and geographic origins.^5^

To contextualize our patient’s atypical presentation and therapeutic challenges, we compared it with an Ethiopian case report describing cutaneous leishmaniasis mimicking borderline-tuberculoid leprosy in 2 immunocompetent males with negative HIV status and favorable response to antimonials. While their cases resembled tuberculoid leprosy, ours displayed lepromatous-like features—including leonine facies and eyebrow alopecia—introducing greater diagnostic complexity. The Ethiopian cases were linked to Leishmania aethiopica, an Old World species, without molecular confirmation, whereas multiple studies have implicated New World species (L. amazonensis, L. mexicana) in diffuse cutaneous leishmaniasis. Despite similar therapy, our patient showed poor clinical improvement, adding therapeutic difficulty. Furthermore, their reliance on biopsy alone contrasts with our multifaceted diagnostic strategy combining histopathology, Ziehl-Neelsen staining, and clinical correlation within resource constraints.^6^

## Conclusion

This case highlights diffuse cutaneous leishmaniasis, mimicking lepromatous leprosy due to leonine facies and eyebrow alopecia. Despite its classical manifestation in New World leishmaniasis, the patient exhibited a poor response to conventional antimonial therapy, necessitating further diagnostic investigations in a resource-limited setting. The exclusion of co-infection with M. leprae through histopathological and microbiological analysis reinforced the final diagnosis. Notably, the patient’s marked clinical improvement following amphotericin B therapy further validated the diagnostic approach. This case underscores the importance of considering atypical presentations of leishmaniasis, particularly when standard treatments fail, and highlights the need for adaptable diagnostic strategies in endemic regions.

## Conflicts of interest

None disclosed.
